# IL-1β primed mesenchymal stromal cells moderate hemorrhagic shock-induced organ injuries

**DOI:** 10.1186/s13287-021-02505-4

**Published:** 2021-08-05

**Authors:** Clotilde Aussel, Nathalie Baudry, Marion Grosbot, Cécile Caron, Eric Vicaut, Sébastien Banzet, Juliette Peltzer

**Affiliations:** 1 Institut de Recherche Biomédicale des Armées (IRBA), 1 rue du Lieutenant Raoul Batany, 92141 Clamart, France; 2 UMR-S-MD 1197, Ministère des Armées et Université Paris Saclay, Villejuif, France; 3 Laboratoire d’Etude de la Microcirculation, UMRS 942 INSERM, University of Paris, Paris, France

**Keywords:** Mesenchymal stromal cells, Priming, Hemorrhagic shock, Immunomodulation, IL-1β, Multiple organ dysfunction syndrome

## Abstract

**Background:**

Organ damages following hemorrhagic shock (HS) have been partly attributed to an immunological dysfunction. The current challenge in the management of HS patients is to prevent organ injury-induced morbidity and mortality which currently has not etiological treatment available. Mesenchymal stromal cells (MSC) are used in clinical cell therapy for immunomodulation and tissue repair. In vitro priming is often used to improve the immunomodulation efficiency of MSC before administration.

**Objective:**

Assess the effect of naive MSC (MSCn) or interleukin (IL)-1β primed (MSCp) treatment in a context of HS-induced organ injury.

**Methods:**

Rats underwent fixed pressure HS and were treated with allogenic MSCn or MSCp. Liver and kidney injuries were evaluated 6h later by histological and biochemical analysis. Whole blood was collected to measure leukocytes phenotypes. Then, in vitro characterization of MSCn or MSCp was carried out.

**Results:**

Plasma creatinine, blood urea nitrogen, and cystatin C were decrease by MSCp infusion as well as kidney injury molecule (KIM)-1 on histological kidney sections. Transaminases, GGT, and liver histology were normalized by MSCp. Systemic cytokines (IL-1α, IL-6, and IL-10) as well as CD80, 86, and PD-1/PDL-1 axis were decreased by MSCp on monocytes and granulocytes. In vitro, MSCp showed higher level of secreted immunomodulatory molecules than MSCn.

**Conclusion:**

An early administration of MSCp moderates HS-induced kidney and liver injury. IL-1β priming improves MSC efficiency by promoting their immunomodulatory activity. These data provide proof of concept that MSCp could be a therapeutic tool to prevent the appearance of organs injury following HS.

**Supplementary Information:**

The online version contains supplementary material available at 10.1186/s13287-021-02505-4.

## Background

Hemorrhagic shock (HS) is an absolute hypovolemia leading to circulatory failure and inadequate perfusion of tissues. Deaths from hemorrhage represents a world health problem, estimated at 1.9 million deaths per year worldwide, 1.5 million of which result from physical trauma [1]. Moreover, in military practice, bleeding is the second leading cause of death after head injury [2]. Twenty-five percent of high-risk polytrauma patients developed post-injury complications ending up with multiple organ dysfunction syndrome (MODS) [3]. During the last decade, many advances have been made in early-phase management strategies; more injured patients survive and reach critical care units. Post-hemorrhagic resuscitation improves tissue perfusion but does not resolve ischemia reperfusion-related damage and activation of the systemic inflammatory response. Indeed, organ injury following trauma-hemorrhage has been attributed to an acquired immunological dysfunction [4, 5] with an imbalance between pro-inflammatory and anti-inflammatory counter-regulatory mechanisms. An excessive pro-inflammatory response (systemic inflammatory response syndrome, SIRS) induced by danger-associated molecular patterns (DAMPs) is characterized by local and systemic release of pro-inflammatory cytokines, complement factors, and coagulation proteins as well as hormonal mediators and excessive activation of innate cells. In parallel, anti-inflammatory mediators are produced, immune cells become anergic, initiating the compensatory anti-inflammatory response syndrome (CARS) [6, 7]. This systemic response aggravates ischemic organ damage. Modulating the immune response could be a potential therapeutic strategy for preventing the complication of trauma-HS as it is in sepsis [8].

Mesenchymal stromal cells (MSC) are multipotent adult stem cells and progenitors isolated from tissues of mesodermal origin such as bone marrow [9]. MSC are defined by the International Society for Cellular Therapy consortium as plastic adherent, expressing positive and negative hematopoietic surface markers, and able to differentiate toward osteoblasts, adipocytes, and chondroblasts [10]. MSC are used in clinical cell therapy for immunomodulation and tissue repair, mediated by direct cellular contact or by paracrine activity [11]. Several evidences of MSC’s therapeutic activity in other SIRS-associated pathology have already been described [12]. Moreover, these cells are highly sensitive to their environment and modifying their culture conditions (priming), for example with inflammatory cytokines, can improve their immunomodulatory efficiency [13]. In several studies, in vitro IL-1β priming was used as a tool to maximize MSC’s immunomodulation [14, 15]. Taken together, there are strong arguments to propose that MSC could protect against HS-induced organ injury, however, to our knowledge, no study evaluated neither naive MSC nor primed MSC therapy in HS models.

The objective of this study was to explore if the occurrence of organ injuries can be prevented in animals undergoing HS by modulating immune system. For this purpose, early administrations of naive MSC (MSCn) or IL-1β-primed (MSCp) have been evaluated.

## Methods

### MSC isolation and culture

Bone marrow was isolated from femurs and tibias of 8 Sprague-Dawley’s rats. MSC were isolated by plastic adhesion, amplified up to 80–90% confluence in a complete medium containing MEM alpha (Biological Industries) supplemented with 10% fetal calf serum (FCS, HyClone) + 1% penicillin streptomycin (Gibco) and 1 ng/mL of bFGF (Peprotech), then pooled at the same ratio for each donor. The cells were frozen in MEMα + 10% Albumin (Vialebex, LFB) + 1% penicillin streptomycin + 10% Dimethylsulfoxide (Sigma Aldrich) and finally preserved in liquid nitrogen. For in vivo experiments, MSC were thawed and seeded at 3000 to 5000 cells/cm^2^. Medium was changed one day after thawing. When MSC reached 90 to 100% of confluence, they were harvested using trypsine-EDTA (Gibco) and suspended in a sterile syringe at a concentration of 2.10^6^ cells/mL of Ringer lactate solution (Fresinus).

### MSC priming

When MSC reached 80% of confluence the medium was changed and replaced by complete medium + 5 ng/mL rat IL-1β (Peprotech) for 24 h. Then, primed MSC were washed twice, harvested, and suspended as described above. The IL-1β priming dose of 5 ng/mL used for our in vivo experiments was chosen following data from the literature but also based on the functional efficiency of MSC primed at 1, 5, or 10 ng/mL in vitro in an immunomodulatory assay (data not shown).

### MSC secreted proteins

After 24 h of priming, MSC’s medium was discarded before performing two washes with PBS. The cells were then incubated in MEM alpha for 1 h at 37 °C. After medium removal, the MSC were again incubated at 37 °C for 24 h in a half the usual volume of alpha MEM to concentrate the secretome. The medium was recovered, centrifuged at 450×*g* for 5 min and the supernatant was stored at − 80 °C. The concentration of several secreted proteins was evaluated by immunoassay: IL-6, IL-1β (R&Dsystems), PGE2 (Abcam), and CCL2 (Abcam).

### Immunomodulatory assay

THP-1 (pro-monocytes ATCC® Manassas, VA, USA) cells were cultured at 280,000 cells/mL in RPMI 1640 Glutamax™ (Gibco, Billings, MT, USA), 10% FBS (Hyclone, San Angelo, Texas, USA), 1% penicillin/streptomycin (Gibco, Billings, MT, USA) and 0.05 mM β-mercaptoethanol (Gibco, Billings, MT, USA). The cells were re-fed every 3 to 4 days. The passages were carried out after 6 to 7 days, the cells were diluted with new medium to reach their conventional seeding density. The production of hTNF-α, hIL-6, and hIL-1RA cytokines was evaluated after stimulation with 1 μg/mL of lipopolysaccharide (LPS) (L6529 from Sigma) of THP-1 co-cultured or not with rat MSC naive or primed by IL-1β (MSCn or MSCp) as described by Zhang et al. [16]. Briefly, THP-1 cells were seeded at 170,000 cells/mL in 1 mL of complete medium in a 6-wells plate, MSC were added at 1:1 to 1:50 ratio, and the co-cultures were exposed to 1 μg/mL of LPS for 24 h (Sigma Aldrich, Saint-Louis, MS, USA). Unstimulated controls were carried out with or without MSC. Supernatants of each condition were then collected and centrifuged, and cytokine levels were evaluated by immunoassay (ELISA Kits, Bio-Techne, Minneapolis, MN, USA). Cytokines concentrations in all conditions were quantified relative to the value obtained in the THP-1 + LPS condition, which had the value 1.

### Animal model

All experimental protocols, procedures, and endpoints criteria were approved by the institutional animal committee of Paris University (French Ministry of Research number CEEALV/#9498).

A total of 64 Sprague-Dawley male rats (Janvier Labs) weighting 392 ± 5 g, were included in this protocol. They were acclimated in a room with a 12-h dark/light cycle for 5 days with free access to water and feeding until they were anesthetized for experiments. The anesthesia was carried out by spontaneous mask ventilation with isoflurane (Iso-Vet, Piramal Healthcare). For induction and during surgery, the FiO_2_ in the mask was 33%; thereafter, it was reduced to 26%. A subcutaneous injection of buprenorphine (50 μg/kg) and 100 μL of local anesthetic, lidocaine hydrochloride (Xylocaine, Aguettant) 2.8 mg/mL + levobupivacaine chlorydrate (Chirocaine, Abbvie) 1.4 mg/mL, was performed on the incision site. These injections were repeated before the animals were awakened at the same dose. Animals were placed on a heating blanket (temperature 37 °C) in supine position. Both carotid artery and jugular vein were catheterized for bleeding the animals (controlled hemorrhage) and for administration of fluid resuscitation, treatment, and blood re-transfusion. After surgery, an injection of 300 μL heparin Choay was carried out (100 UI/mL, Sanofi).

### Experimental protocol (Fig. 1A)

Forty-six rats were allocated to four groups as described below:

**Fig. 1:**
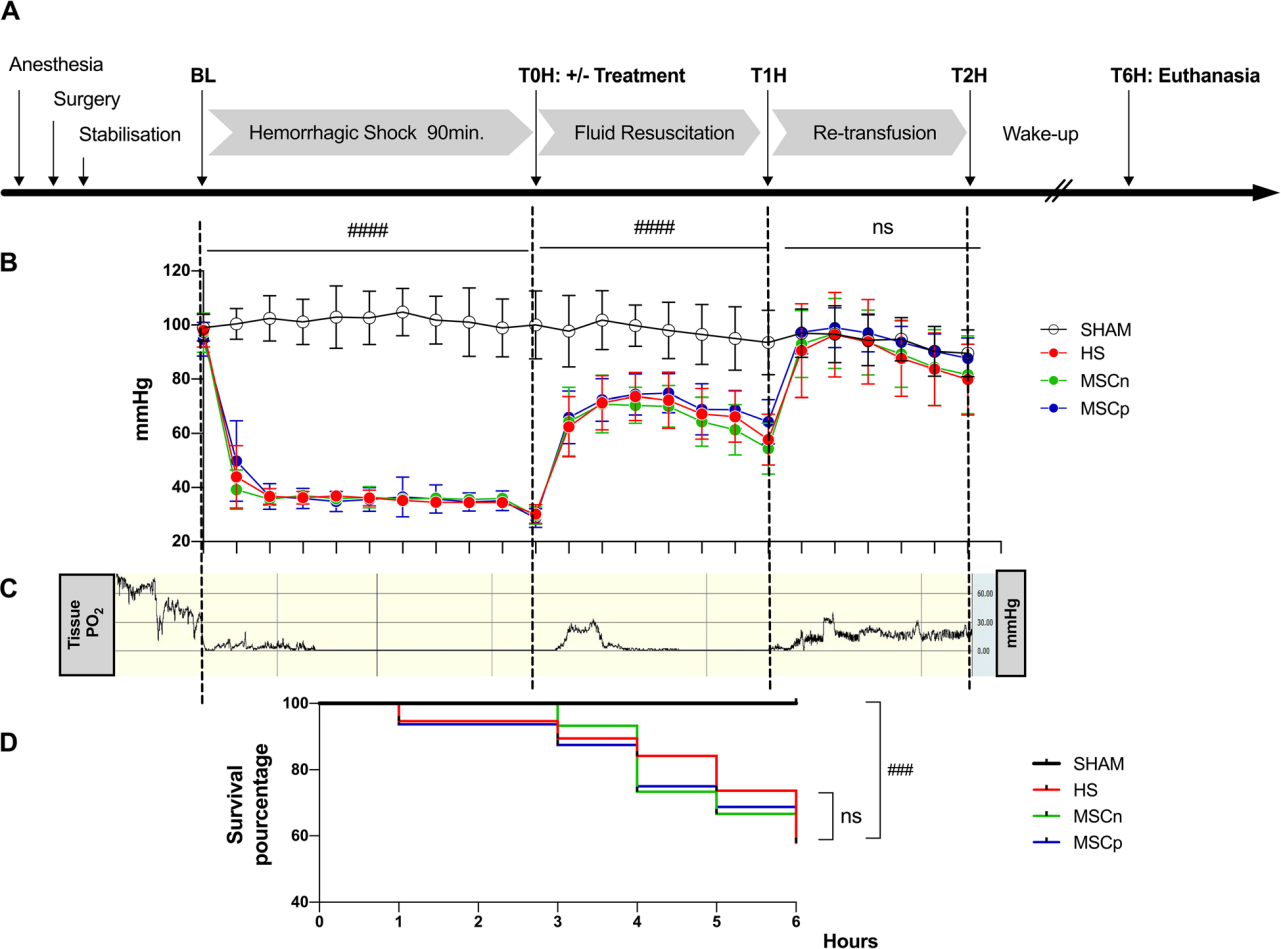
Characteristics of the animal model. **A** Synopsis of the experiment. Rats were subjected to controlled pressure hemorrhage for 90 min. After 1.5 h of shock (T0H), the hemorrhagic rats were resuscitated with a lactate ringer infusion (with or without MSC treatment) followed by re-transfusion of spoliated blood. At T6H, the animals were sacrificed and the samples collected. **B** Mean blood pressure over time in sham, untreated hemorrhagic (HS) and hemorrhagic treated with MSCn and MSCp groups of animals, during the phase of hemorrhagic shock, fluid resuscitation, and re-transfusion followed by SEM (*n* = 10, 14, 10, and 12 in sham, HS, MSCn, and MSCp groups respectively). The 3 phases of the protocol were analyzed separately by non-parametric analysis of longitudinal data in factorial experiments (F1-LD-F1) follow by a pair comparison test, ^####^*p* < 0.001. **C** Individual curve of tissue oxygen tension (tPO_2_) in the renal cortex during hemorrhagic shock, fluid resuscitation and re-transfusion in an untreated HS rat. **D** Kaplan-Meier survival curve after 90 min of bleeding at 35 ± 2 mmHg were analyzed through stat Log-rank ^###^*p* < 0.005 (*n* = 10, 19, 15, and 16 in sham, HS, MSCn, and MSCp groups respectively)

*Sham group*. Rats underwent surgery without bleeding, fluid resuscitation, and blood re-transfusion. They received 0.9% saline (2 mL/h) after anesthesia.

*Hemorrhage shock group (HS)*. Blood was withdrawn to reach a 35 ± 2-mmHg MAP (mean arterial pressure) and maintained for 90 min. At T0H, Ringer lactate infusion started (2 mL/kg/min) equal to twice the volume of spoiled blood. After 1 h (T1H), blood withdrawn in heparinized (25 UI) syringes was re-transfused.

*Treated groups with naive MSC (MSCn) or IL-1β-primed MSC (MSCp)*. At T0H after hemorrhagic shock, MCSs were slowly injected intravenously into the flow of Ringer lactate (10^6^ cells). Blood withdrawal was re-transfused at T1H.

For all animals, the catheters were removed at T2H, the skin sutured and then the isoflurane was stopped. At the time of sacrifice (T6H), samples of blood and organs (kidney, liver, lung) were taken.

Fourteen animals died before T5H and were excluded from biological analyzes (5 HS, 5 MSCn, and 4 MSCp). However, they were included in the Kaplan-Meier survival analysis.

### Tissue oxygen pressure measurement

Four additional rats were used to measure tissue oxygen pressure (tPO_2_) in the renal cortex (2 sham and 2 HS) and were sacrificed at T2H without being awakened. The tPO_2_ was measured with an optical fiber oxygen measurement system (OxyMicro; WPI, Hitchin, UK). The rats were lying on their side. The left kidney was exposed through a midline incision. A needle containing the microsensor’s optical fiber (MicroTip 501656, WPI, Hitchin, UK) was introduced tangentially under the renal capsule using a micromanipulator. The microsensor was extended into renal cortex (3 mm). Kidney was covered with transparent film to avoid desiccation. The tPO_2_ was continuously recorded at 1-s intervals for 210 min.

### Blood gas, glycemia, and lactatemia

Blood tests were performed at three experimental time-points: before (baseline, BL), at the end of the hemorrhage (T0H), and at T6H on the 46 animals. Blood samples were collected from the arterial catheter to measure arterial blood gases (Rapidlab 348 EX, Siemens HealthCare), glycemia (Verio, OneTouch), and lactatemia (THE EDGE, ApexBio). The PaO_2_/FiO_2_ ratio was the calculated by dividing the arterial oxygen level by the fraction of inspired oxygen measured on the ventilation mask. Alveolar-arterial gradient is a measure by the difference between the alveolar oxygen concentration (calculated from the blood gas data) and the arterial oxygen concentration (measured from the blood gases).

### Plasma analysis

Whole blood collected from the aorta at T6H on heparinized syringe was centrifuged at 2600×*g* during 12 min. Plasma concentrations of cytokines, IL-1β, IL-1α, IL-6, and IL-10 (Duoset, R&D) as well as cystatin C (Quantiquine R&D), were measured in duplicates using sandwich ELISA essay according to the manufacturer’s instructions.

Plasma biochemistry analyses were performed using a Cobas 6000 automated analyzer in Percy military hospital (Clamart, France). Liver function was assessed by plasma levels of aspartate aminotransferase (AST), alanine aminotransferase (ALT), and gamma-glutamyl transferase (GGT). Kidney function was assessed by the plasma level of blood urea nitrogen (BUN) as well as the plasma levels of creatinine.

### Histological and immunohistochemistry

The preparation of slides and stains was carried out by the histology department of the Cochin Institute (HistIM; INSERM U1016, CNRS UMR 8104, University of Paris). Briefly, the organs were fixed 48–72 h in 4% buffered formaldehyde, transferred to 70% ethanol, and embedded in paraffin. Sections were then cut into 4μm slices for hematoxylin-eosin-safran (HES) (liver) and periodic acid-Schiff (PAS) (kidney) staining. Slides were scanned and examined on CaseViewer software by two blind investigators. Kidney Tubular injury score (tubular dilation, tubular cast formation, and tubular necrosis) [17] as quantified in the external medulla. Liver injury score (congestion, vacuolization, and necrosis of hepatocytes) [18] was quantified on 30 randomly selected fields. Lung injury score included leukocytes in the interstitial or alveolar space, proteinaceous debris filling the airspaces, and alveolar septal thickening [19] and was quantified on total section scanning. For kidney injury molecule-1 (KIM-1), sections were labeled with goat anti-rat KIM-1 antibody at 1-μg/mL concentration (R&D). Goat anti-rat IgG (R&D) was used as a negative control. The immunoreactivity was visualized using the tissue staining kits reagent (Dako). Slides were scanned and 5 randomly selected fields of the kidney external medulla were analysis with Fiji software. For each kidney sample, 5 fields were digitized on the negative control slides and transferred to the KIM-1 slides. A fixed threshold was applied to all images to estimate the percentage of positive DAB labeling. The difference between this positive DAB labeling (KIM-1 slides minus controlled Ig) represents the KIM-1 labeling of the renal tubules expressed as a percentage of area.

### Cytometry analysis

For phenotype characterization of white blood cells, a multi-parameter analysis was carried out by flow cytometry (Navios, Beckman Coulter®). Whole blood was collected in tube field with cellular antigen stabilization solution (Transfix, Cliniscience) and kept at 4 °C up to 14 days. Red blood cells were lysed with Macs lysing solution. To minimize non-specific antibody binding, after centrifugation, cells were incubated in a buffer containing PBS, 2% human albumin (LFB), and 2 μg/mL polyvalent human immunoglobulin (R&D Systems) before staining. Cells were then incubated with fluorescent monoclonal antibodies: CD8a (OX8, eBioscience), CD4 (OX35, eBioscience), CD3 (1F4, BioRad), CD80 (3H5, BioRad), CD279 or PD-1 (KLH, Bioss), CD274 or PD-L1 (KLH, Bioss), CD11b/c (REA, Miltenyi), CD28 (REA, Miltenyi), CD45 (REA, Miltenyi), CD86 (REA, Miltenyi), and MHC2 (REA, Miltenyi), at a saturating concentration for 20 min at + 4 °C. Isotype antibodies, non-stain cells, and fluorescence minus one were used as controls. The FlowJo software was used to set up gating and analyze positivity frequencies of the makers of interest.

### Statistical analyses

Mean arterial pressures are represented as mean ± SD. During the three phases (hemorrhage, fluid resuscitation, and re-transfusion), the changes in mean arterial pressure between groups over time were analyzed by non-parametric ANOVA for longitudinal data (F1-LD-F1, in R software, R 3.3.2, http://www.r-project.org) followed by a pair comparison test. Survival analysis represented in a Kaplan-Meier survival curve was analyzed with a Log-rank test (Mantel-Cox) using R software. All other data are presented as median ± interquartile range and statistical analyses were performed on the Prism 8 Graphpad software. Mann-Whitney statistical analysis was used to test the effect of our hemorrhaging model versus sham. Kruskal-Wallis statistical analysis with Dunn’s correction (adjust for multiplicity) was used to test the effect of our treatments (MSCn and MSCp) to the HS group. The significance level was defined as a *p* value < 0.05. In the immunomodulatory assay, all MSC coculture conditions were analyzed versus THP-1+LPS with Kruskal-Wallis statistical analysis with the adjustment of Dunn’s (Prism 8 Graphpad software).

## Results

### Characteristics of the animal model

Prior to the hemorrhagic shock phase, MAP, blood gas, glycemia, and lactatemia analysis were not statistically different between experimental groups (data not shown). As shown in Fig. 1B, the evolution of MAP over time during the three phases (hemorrhagic shock, fluid resuscitation, and re-transfusion) was similar across hemorrhagic groups. During the hemorrhagic shock phase, the median of the 3 hemorrhagic groups MAP were significantly reduced compared to the sham group and decreased from 101 [IQR 14] to 36 [IQR 4] mmHg (Fig. 1B, *p* < 0.0001). The blood volumes collected to achieve this MAP target were 39 [IQR 4] mL/kg, 42 [IQR 5] mL/kg, and 41 [IQR 6] mL/kg in the HS, MSCn and MSCp groups respectively and were not significantly different between the hemorrhagic groups (treated or not). With fluid resuscitation, MAP tended to increase but remained significantly lower than in the sham group. After blood re-transfusion in the third phase, the MAP of the hemorrhagic groups were restored and were no longer significantly different from that of the sham group.

During hemorrhagic shock phase, tissue oxygen pressure (tPO_2_) measured in the renal cortex decreased rapidly from 50 to 2 mmHg with the drop in MAP (Fig. 1C). During fluid resuscitation, tPO_2_ rose transiently before falling back to values close to zero. On the other hand, blood re-transfusion allowed tPO_2_ to rise and stabilize in the renal cortex. The tPO_2_ analysis was used as a control of the model and not as an experimental data. Therefore, the measurements were only performed on four rats (2 sham and 2 HS); no statistical analysis was performed on these results.

Survival analysis showed a significant increase in mortality in the 3 HS groups compared to the sham group with 57.9, 66.7, and 68.7% survival in the HS, MSCn, and MSCp groups respectively vs 100% in the sham group (Log-rank test *p* = 0.005, Fig. 1D). The treatments did not show a significant effect on survival at T6H.

### Blood gas, glycemia, and lactatemia

Blood withdrawal (T0H) induced metabolic acidosis with a significant decrease in pH, base excess, and bicarbonates (overall median of hemorrhagic groups 7.29 [IQR 0.09], − 11.0 [IQR 4.28], and 15.7 [IQR 4.40] mmol/L respectively) associated with a significant increase in lactate concentration, which peaked at 12.0 [IQR 3.6] mmol/L for the 3 hemorrhagic groups (Table 1). Reactive hyperventilation resulted in a decrease in PaCO_2_ associated with an increase in PaO_2_ (overall median of hemorrhagic groups 35 [IQR 8.6] and 140 [IQR 18.2] mmHg respectively). Hemorrhagic shock also induced a decrease in hematocrit (overall median of hemorrhagic groups 25 [IQR 5] %) and an increase in blood glucose (overall median of hemorrhagic groups 244 [IQR 161] mg/dL) at T0H. At T6H, overall acid-base results were normalized in the hemorrhagic animals (treated or untreated) with no difference with the sham group. Hematocrit was no longer significantly different between groups, and only blood glucose levels remained significantly higher in animals treated with MSCp.

**Table 1:** Blood gases, lactatemia, and glycemia

		Sham	HS	MSCn	MSCp
**T0H**	**pH**	7.39 (0.08)^####^	7.31 (0.09)	7.31 (0.14)	7.28 (0.07)
	**PaO**_**2**_ **(mmHg)**	104 (31.3)^####^	142 (16.4)	138 (19.2)	136 (32.1)
	**PaCO**_**2**_ **(mmHg)**	45.3 (8.6)^###^	34.9 (7.4)	32.7 (9.4)	37.9 (13.2)
	**BE**	2.30 (2.78)^####^	− 11.00 (5.53)	− 11.00 (4.33)	− 11.00 (3.43)
	**HCO**_**3**_^**−**^ **(mmol/L)**	27.1 (3.3)^####^	15.7 (5.00)	15.5 (2.55)	16.2 (4.2)
	**Ht (%)**	40 (6.3)^####^	26 (3.5)	25 (5.0)	23 (5.8)
	**Lactate (mmol/L)**	1.9 (1.25)^####^	12.9 (2.28)	11.1 (5.11)	11.0 (4.00)
	**Glucose (mg/dL)**	138 (54.3)^#^	210 (153.0)	280 (264.5)	245 (117.5)
**T6H**	**pH**	7.45 (0.05)	7.41 (0.20)	7.41 (0.10)	7.39 (0.07)
	**PaO**_**2**_ **(mmHg)**	96 (26.0)	102 (25.5)	113 (45.0)	105 (41.9)
	**PaCO**_**2**_ **(mmHg)**	38.5 (5.0)	40.4 (12.0)	38.9 (8.5)	44.6 (9.5)
	**BE**	2.80 (3.80)	2.40 (5.30)	1.70 (5.33)	1.60 (4.00)
	**HCO**_**3**_^**−**^ **(mmol/L)**	26.3 (2.65)	26.4 (3.5)	25.2 (5.02)	27.0 (3.70)
	**Ht (%)**	32 (3.5)	29 (8.0)	29 (5.5)	31 (5.0)
	**Lactate (mmol/L)**	1.8 (1.35)	2.5 (8.05)	1.55 (1.45)	1.5 (2.3)
	**Glucose (mg/dL)**	131 (40.0)	138 (51.0)	129 (45.5)	162 (33.0)*

Blood gases, lactatemia, and glycemia before treatment injection (T0H) and at the end of the experimental period (T6H) in sham, untreated hemorrhagic (HS), and hemorrhagic treated with naive MSC (MSCn) and priming MSC (MSCp) groups of animals. Data are expressed as median and interquartile range. Sham, MSCn, and MSCp were compared to HS group: *p < 0.05, **p < 0.01, ***p < 0.005, or ****p < 0.001 with a Dunn’s test (with adjustment for multiplicity)

### MSCp attenuated hepatic injury after HS

**Fig. 2:**
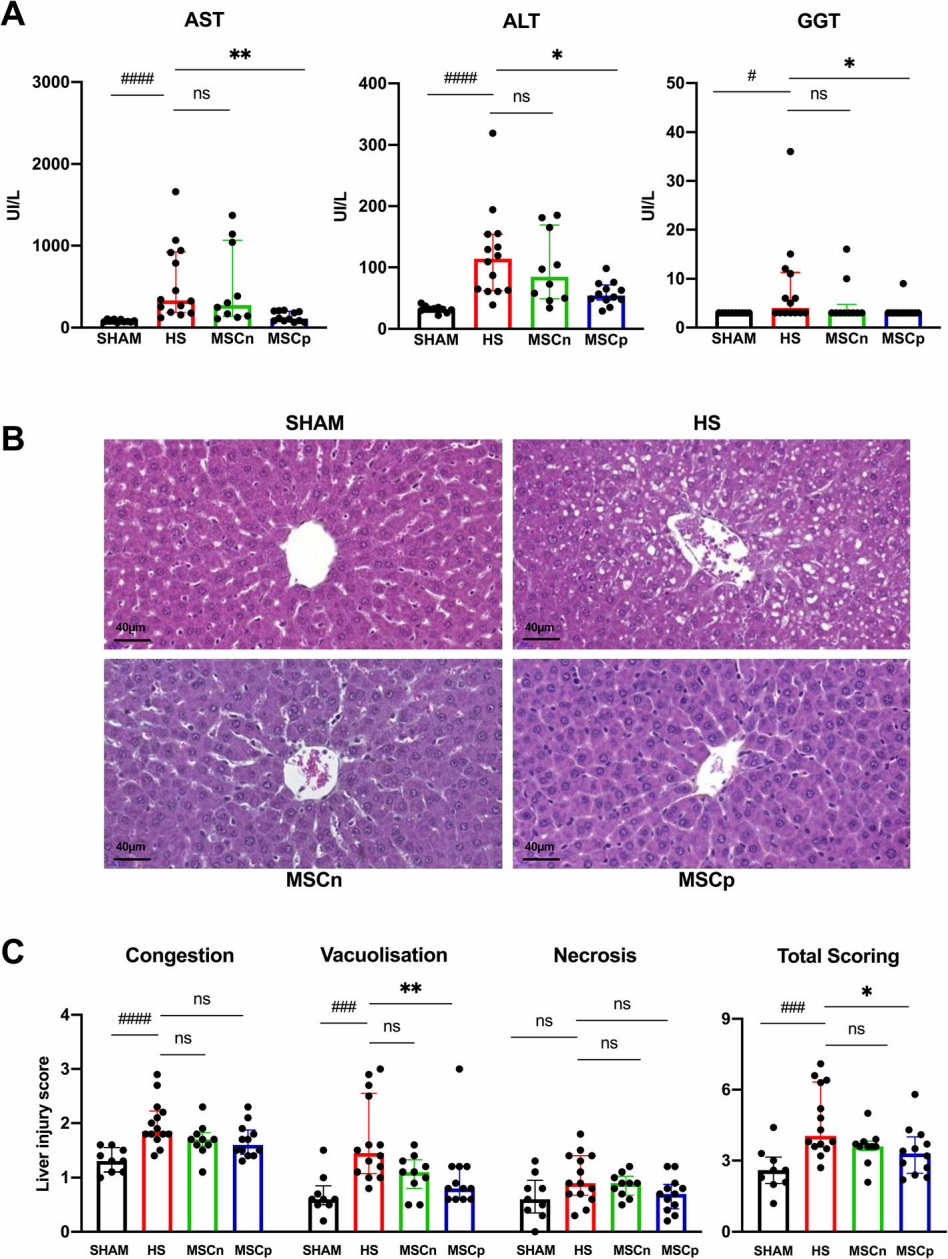
Hepatic injury. **A** Plasma AST, ALT, and GGT levels (*n* = 10, 14, 10, and 12 in sham, HS, MSCn, and MSCp groups respectively). **B** The liver was collected and stained by HES. Representative pictures are presented for every experimental group. **C** Liver injury scores (*n* = 9, 14, 10, and 12 in sham, HS, MSCn, and MSCp groups respectively). Data are expressed as median and interquartile range in the four experimental groups. Mann-Whitney statistical analysis was used to test the effect of our hemorrhaging model versus sham, ^#^*p* < 0.05, ^###^*p* < 0.005, or ^####^*p* < 0.001. Dunn’s test (with adjustment for multiplicity) was used to compare the effects of the treatments (MSCn and MSCp) to the HS group, *p < 0.05 or **p < 0.01

Hemorrhagic shock resulted in liver injury visible at T6H (Fig. 2). Plasma levels of AST, ALT, and GGT significantly increased in the HS group compared to the sham group (Fig. 2A). The evaluation of HS hepatic histological sections also revealed evidence of congestion, vacuolization, and hepatocyte necrosis (Fig. 2B, C). Injection of MSCp significantly decreased AST levels (334 [IQR 736] vs 111 [IQR 119] UI/L for HS and MSCp respectively, *p* < 0.0014) and GGT levels (4.00 [IQR 8.20] vs 3.00 [IQR 0.00] UI/L for HS and MSCp respectively, *p* < 0.0432) (Fig. 2A) and reduced the total score for histological lesions in the liver compared to the untreated group (HS) (*p* < 0.0214) (Fig. 2B, C). On the other hand, MSCn treatment did not significantly prevent hepatocellular injury (Fig. 2).

### MSCp attenuated kidney injury and dysfunction after HS

**Fig. 3:**
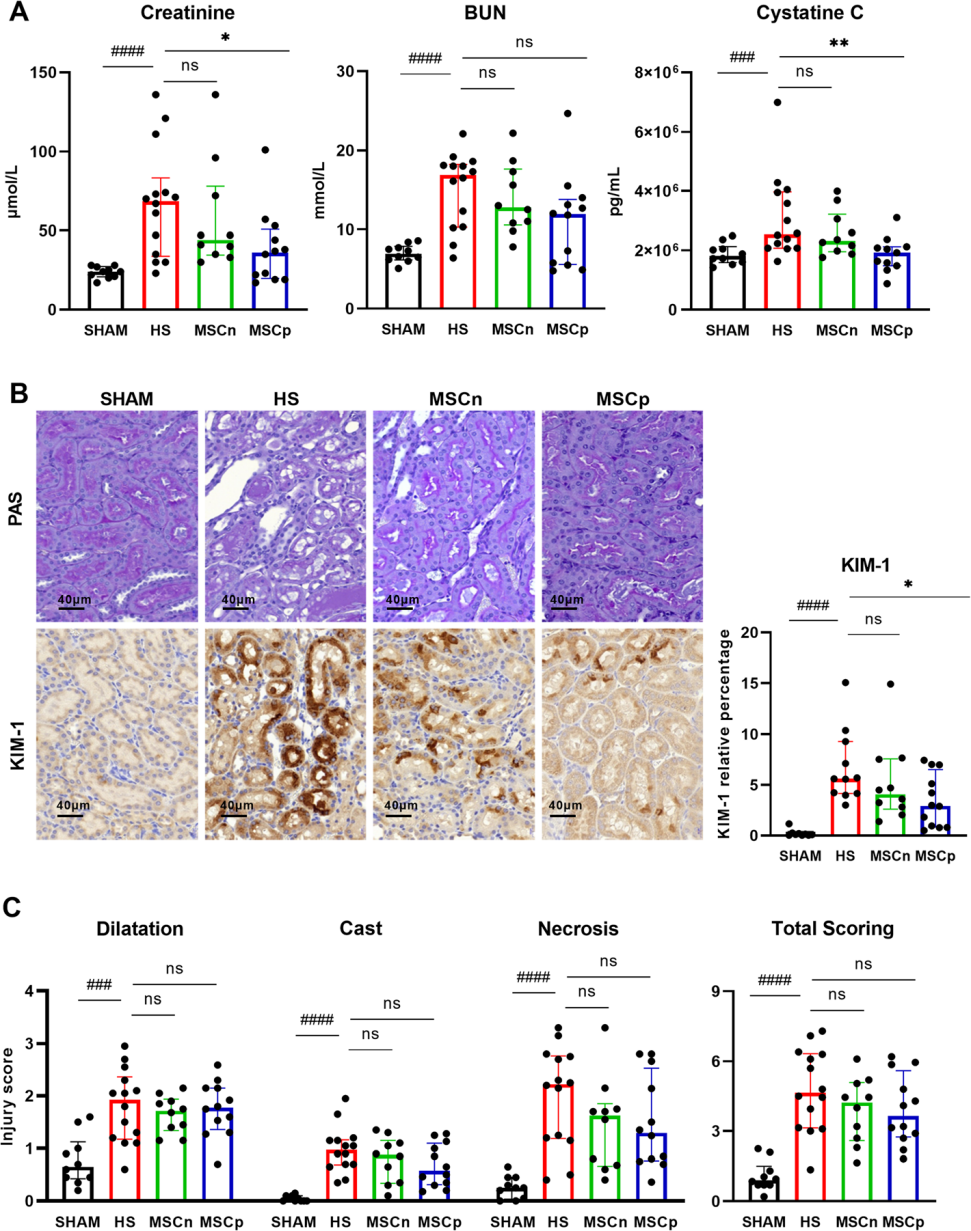
Kidney injury and dysfunction. **A** Plasma levels of creatinine, BUN, and cystatin C (*n* = 10, 14, 10, and 12 in sham, HS, MSCn, and MSCp groups respectively for creatinine and BUN; *n* = 10, 14, 10, and 11 in sham, HS, MSCn, and MSCp groups respectively for cystatin C). **B** The kidney was harvested and stained with PAS and immunohistochemistry for KIM-1. Representative images of the tubules of the renal external medullary pictures are presented for each experimental group. **C** Tubular injury scores (*n* = 10, 14, 10, and 12 in sham, HS, MSCn, and MSCp groups respectively). KIM-1 quantification by immunohistochemistry (*n*= 10, 11, 10, and 12 in sham, HS, MSCn, and MSCp groups respectively for KIM-1 immunohstochemistry). Data are expressed as median and interquartile range in the four experimental groups. Mann-Whitney statistical analysis was used to test the effect of our hemorrhaging model versus sham, ^###^*p* <0.005. or ^####^*p*<0.001. Dunn’s test (with adjustment for multiplicity) was used to compare the effects of the treatments (MSCn and MSCp) to the HS group, *p value < 0.05 or **p < 0.01

HS resulted in an acute kidney injury (AKI) defined as a significant increase in plasma creatinine, urea (BUN), and cystatin C levels compared to the sham group (Fig. 3A). Furthermore, rats undergoing HS showed typical but scattered acute tubular necrosis in the outer medulla of kidney section evaluated by the presence of dilatation with epithelial cells necrosis at the proximal tubules, sometimes associated with the presence of cast in the lumen of these tubules (Fig. 3B, C). Similarly, KIM-1 expression was upregulated after HS (Fig. 3B, C).

The administration of MSCn had no significant effect on AKI plasma markers compared to the HS group. However, MSCp administration significantly improved renal function, with lower levels of creatinine (68.5 [IQR 49.5] vs 36.0 [IQR 31.0] μmol/L for HS and MSCp respectively, *p* < 0.0311) and cystatin C (2.54 [IQR 1.92] 10^6^ vs 1.92 [IQR 0.64] 10^6^ pg/mL for HS and MSCp respectively, *p*< 0.0061) compared to the HS group. BUN remained non-significantly different between the MSCp and HS groups (Fig. 3A). Moreover, the administration of MSC (naive or primed) tended to improve the scoring of tubular necrosis (Fig. 3C). KIM-1 mRNA level and histochemistry staining were lower after in MSCp group (Fig. 3C).

### MSC or HS had no effect on lung histology and ventilation

No ventilation parameters were dysregulated by HS or MSC infusion. The PaO_2_/FiO_2_ ratio remained greater than 300 mmHg in all groups, meaning that the rats were not hypoxemic. No difference was found between groups for PaO_2_/FiO_2_ ratio, as well as for the alveolar-arterial gradient*.* The histological evaluation of lung sections revealed no evidence of diffuse alveolar damage evaluated by leukocyte infiltrate, proteinaceous debris filling the airspaces, and alveolar septal thickening (Fig. S1).

### MSCp reduced plasma cytokines concentration

**Fig. 4:**
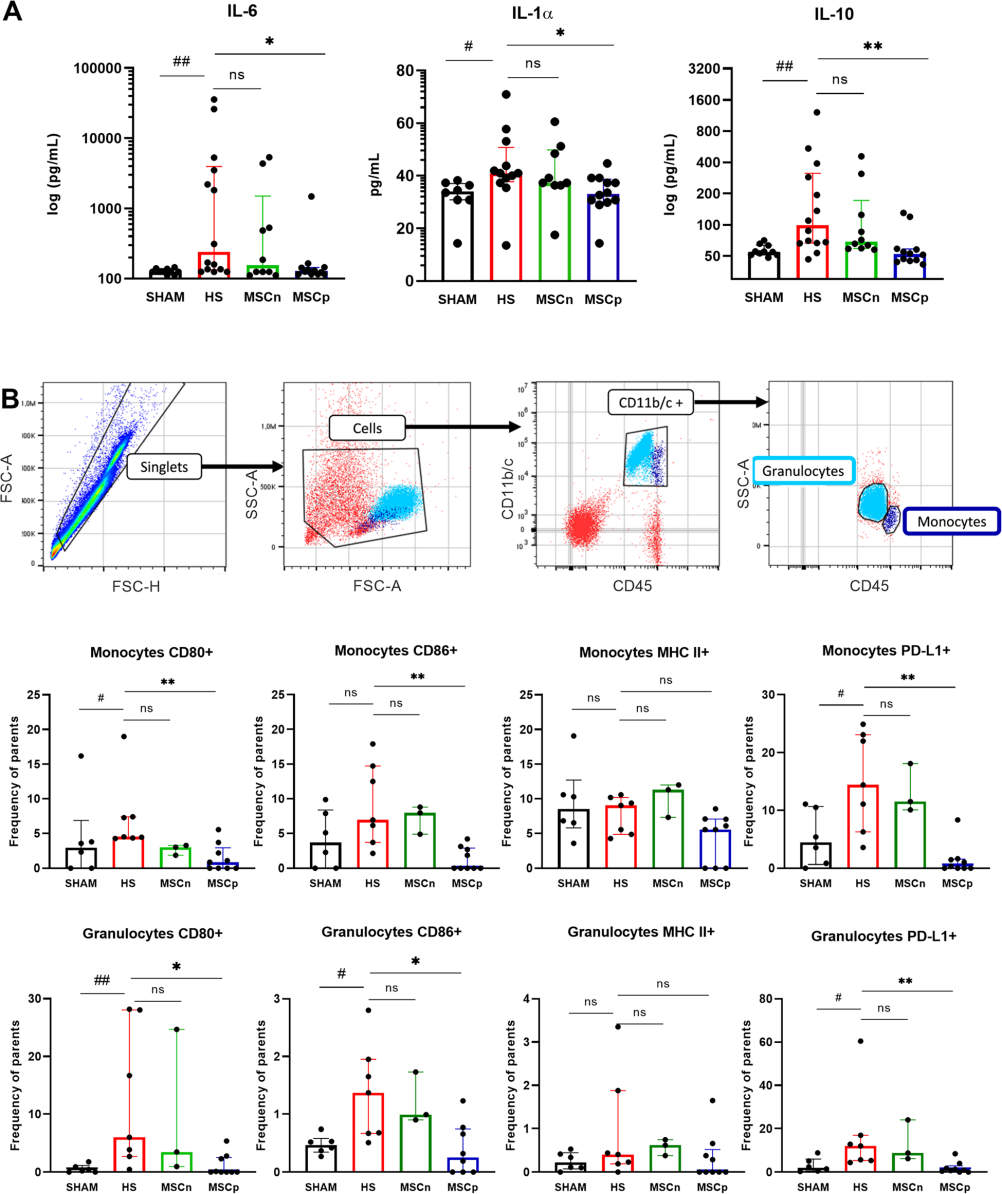
Systemic inflammation. **A** Circulating concentrations of anti and pro-inflammatory cytokines IL-6, IL-1α, and IL-10 (*n* = 10, 13, 10, and 12 in sham, HS, MSCn, and MSCp groups respectively). **B** At the top, representative histograms of flow cytometry gating strategies. Following identification of single cells, the debris was eliminated. The CD11b/c+ CD45+ population were selected and then, monocytes or granulocytes were separated upon their side squatter and CD45 characteristics. Below, frequency of monocytes parents’ positives cells were expressed for CD80, CD86, MHC II, and PD-L1 markers (*n* = 6, 7, 3, and 9 in sham, HS, MSCn, and MSCp groups respectively). Data are expressed with interquartile range. Mann-Whitney statistical analysis was used to test the effect of our hemorrhaging model versus sham, ^#^*p* < 0.05, ^##^*p* < 0.01. Dunn’s test (with adjustment for multiplicity) was used to compare the effects of the treatments (MSCn and MSCp) to the HS group, *p value < 0.05 or **p < 0.01.

Because HS induced a systemic inflammatory response, we measured plasma concentrations of the IL-1β, IL-1α, IL-6, and IL-10 cytokines. At T6h, circulating levels of IL-1α, IL-6, and IL-10 in HS animals were increased compared to sham rats. MSCn administration induced no difference between groups, while MSCp decreased all cytokines concentration (IL-1α, 41 [IQR 13] vs 33 [IQR 9] pg/mL for HS vs MSCp, *p* < 0.0222; IL-6, 241 [IQR 3822] vs 128 [IQR 28] pg/mL for HS vs MSCp, *p* < 0.0257; and IL-10, 98.8 [IQR 245] vs 52.3 [IQR 13.5] pg/mL for HS vs MSCp, *p* < 0.0016) (Fig. 4A). IL-1β was undetectable in all animals except 4 HS and 2 MSCn (data not shown).

### MSCp decreased co-receptors expression on monocytes and granulocytes

The monocyte population was identified as CD45+ CD11b/c+ and low granularity whereas granulocytes were CD45+ CD11b/c+ and high granularity (Fig. 4B). HS induced a minor change in monocytes phenotype; CD80 and PD-L1 were higher after HS than in sham animals. Monocytes antigen presentation co-stimulation receptors CD80 and CD86 were strongly decreased by MSCp infusion (CD80, 4.52 [IQR 3.07] vs 0.84 [IQR 2.93] % positive cells in HS and MSCp respectively, *p* < 0.0022; CD86, 6.95 [IQR 11.0] vs 0 [IQR 2.89] % positive cells in HS and MSCp respectively, *p* < 0.0047). The inhibition marker PDL-1 was also decreased by MSCp (PDL-1, 14.4 [IQR 16.8] % vs 0.32 [IQR 1.53] % in HS and MSCp respectively, *p* < 0.0022). MHC II was not modulated on monocytes by any protocol (Fig. 4B). Similar results were found in granulocytes, with an increase expression of CD80, CD86, and PD-L1 in HS group compared to sham animals. Granulocytes antigen presentation co-stimulation receptors CD80 and CD86 were strongly decreased by MSCp infusion (CD80, 6.05 [IQR 25.35] vs 0.00 [IQR 2.55] % positive cells in HS and MSCp respectively, *p* < 0.01; CD86, 1.37 [IQR 1.28] vs 0.25 [IQR 0.74] % positive cells in HS and MSCp respectively, *p* < 0.0272). The inhibition marker PDL-1 was also decreased by MSCp (PDL-1, 12.1 [IQR 11.65] % vs 0.87 [IQR 2.89] % in HS and MSCp respectively, *p* < 0.006). MHC II was not modulated on monocytes and granulocytes by any protocol (Fig. 4B).

### IL-1β priming stimulated the immunomodulatory properties of MSC

First, we verified that rat bone marrow MSC fulfill the minimal criteria defining MSC [10]. Rat MSC adhere to plastic, proliferate, and expressed the classical surface markers CD29, CD73, CD90, and CD105 and were negative for CD11b, CD45, and HLA-DR. They also differentiate into osteoblasts, chondroblasts, and adipocytes in vitro*.* Finally, formulation for in vivo experiments (Ringer lactate suspension) and storage before administration (+ 4 °C) have been set up to optimize the best cell viability (data not shown).

**Fig. 5:**
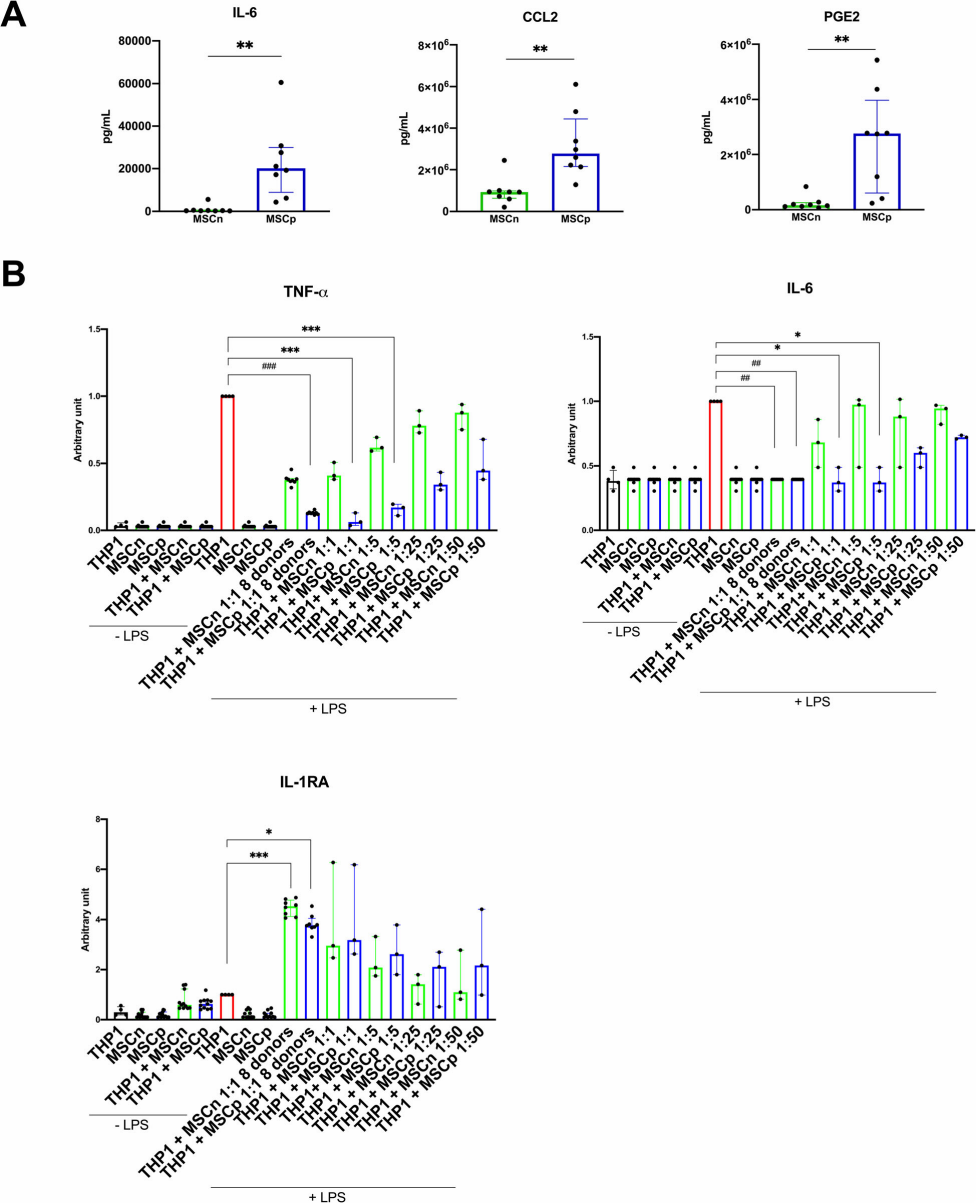
IL-1β priming stimulate immunomodulatory property of MSC. **A** Concentration of IL-6, CCL2, and PGE2 (*n* = 8 and 8 in MSCn and MSCp groups respectively). Data are expressed with interquartile range. Mann-Whitney statistical analysis was used, **p < 0.01. **B** TNF-α, IL-6, and IL-1RA secretion levels from the LPS-activated THP-1 coculture with or without rat MSC naive (MSCn) or primed by 5 ng/mL of IL-1β (MSCp) from a pool of 8 donors or individual donors (MSCn or p 8 donors) at the ratio 1: 1 (1 MSC for 1 THP-1) up to a ratio of 1:50 were evaluated. The data are expressed as the median ± interquartile (*n* = 8 donors or 3 MSC replicates from the pool). Friedman’s tests for matched data (donor MSCn vs MSCp 8 donors) or Kruskal-Wallis for unpaired data (THP-1 vs MSC, and donors of MSC vs pool of MSCn, and MSCn vs MSCp) with Dunn’s correction were applied to assess the significance of observed differences *p < 0.05, **p < 0.01 or ^##^*p* < 0.01, ^###^*p* < 0.005

IL-1β priming stimulated immunomodulatory properties of MSC. The ability of MSCp to produce soluble active molecules was examined. IL-6 and CCL2 protein concentrations were upregulated in MSCp culture supernatants (IL-6, 3.35 [IQR 2.66] 10^2^ pg/mL vs 202 [IQR 210] 10^2^ pg/mL, *p* < 0.0078; CCL2, 9.37 [IQR 3.58] 10^5^ pg/mL vs 27.7 [IQR 22.8] 10^5^ pg/mL for MSCn and MSCp respectively, *p* < 0.0078) (Fig. 5A). PGE2 was also significantly increased in MSCp supernatants (1.65 [IQR 1.3] 10^5^ pg/mL vs 27.6 [IQR 33.7] 10^5^ pg/mL for MSCn and MSCp respectively, *p* < 0.0078) (Fig. 5A). Finally, the efficacy of MSC-IL-1β priming was evaluated in a functional test of inflammation using monocytic cell line (THP-1). The anti-inflammatory capacities of the MSC from the pool (used for the in vivo experiments) as well as the different donors separately were compared. MSCp, but not MSCn, induced a significant decrease in the concentration of TNF-α at the ratio 1:1 (1 [IQR 0] vs 0.129 [IQR 0.013] for THP-1 LPS vs THP-1 LPS MSCp). A decrease was also observed by the coculture of THP-1 + MSCp pool at the ratio 1:1 and 1:5 (1 [IQR 0] vs. 0.0627 [IQR 0.094] or 0.170 [IQR 0.086] for THP-1 LPS vs. THP-1 LPS MSCp 1:1 or 1:5). The presence of MSCn and MSCp only induced a tendency to increase IL-1RA compared to THP-1 + LPS condition (Fig. 5B). Moreover, no significant difference was observed for TNF-α or IL-6 between coculture conditions with THP-1-MSC or with the pool of MSC donors, whether naive or primed after LPS stimulation (Fig. 5B). However, a significant difference in IL-1RA concentration was found between the THP-1 + LPS and THP-1 + LPS + MSC 8 donors, but not of the pool (1 [IQR 0] vs 4.53 [IQR 0.646] or 3.77 [IQR 0.362] for THP-1 LPS vs THP-1 LPS MSCn donors or THP-1 LPS MSCp donors) (Fig. 5B). Unstimulated cells or LPS-stimulated MSC alone (used as control samples) had an undetectable or very low threshold for the 3 cytokines assayed (TNF-α, IL-6, and IL-1RA). Finally, it is important to note that IL-1β was undetectable in MSC supernatant after 24 h of culture.

## Discussion

In this report, we showed that IL-1β-primed MSC attenuated HS-induced early organ injury and dysfunction and reduced the SIRS/CARS syndrome as shown by a decrease in plasma cytokine concentrations and phenotypic activation of circulating CD11bc+ cells.

The fixed pressure hemorrhagic shock model in Sprague-Dawley rats chosen in this study allows a better control of the hypotension and of the volume of blood loss. Moreover, it allows to use fewer animals by limiting the risk of mortality compared to uncontrolled hemorrhage models [20]. The key points of our experimental protocol (spoliation time, vascular filling volumes, blood transfusion…) were based on existing protocols in the literature [21–24]. The fixed pressure hemorrhagic shock model established was extremely severe as demonstrated by lactate level [25] and led to a very high mortality at 24 h with serious and irreversible organ damages. Therefore, we decided to choose the analysis time at T6H post-hemorrhage to avoided unbalance in groups due to early mortality. Indeed, in the literature, the time for the first blood sampling of patients (from emergency departments or intensive care units) vary considerably between different studies, making it difficult to interpret the kinetics of the onset of dysregulation or injuries. This is why the knowledge of immunity changes during the ultra-early post-injury phase (particularly within the first hour) is still limited [26]. Therefore, we thought that the early observation time is a good option to explore hypotheses on the driver mechanisms leading to further organ injuries. At T6H, we observed kidney and liver injuries as previously reported by Rönn et al. in a similar model [23]. However, neither ventilation parameters nor lung histological scoring were affected by HS contrary to what has been shown by others [27]. It can be argued that these authors have a more severe model of HS. Another hypothesis, as proposed previously, could be that the blood flow has been redistributed at the expense of the splanchnic, skeletal muscles, and skin circulations [6] in order to preserve the circulation in the vital organs including the lungs. Moreover at 6 h post HS immunohistochemical tissue labeling with CD45 or CD68 showed no evidence of kidney, liver, or lung infiltration of leukocytes.

It is important to note that we administered 1.10^6^ MSC to each rat of approximately 350 to 400 g, i.e. 2.5–3.10^6^ MSC/kg, corresponding to doses compatible with clinical practice. Indeed, the amounts of MSC used in animal studies are often much greater than doses used in humans, this could bias the therapeutic effect observed as well as the understanding of the mechanisms of action of MSC. We observed that MSCp infusion attenuated the early AKI and liver injury. MSCp administration tended to moderate tubular cell necrosis and significantly decrease KIM-1 in tubulars cells, a biomarker of renal proximal tubule injury [28]. MSCp also attenuated hepatocytes vacuolization and normalized transaminase plasmatic levels markers of liver injury. It was previously demonstrated that MSC therapy is effective in reducing acute kidney injury (AKI) or liver injury in diverse experimental models of isolated organ damage including ischemia/reperfusion (I/R) or hepatotoxic and nephrotoxic drug [29, 30]. MSC are also currently evaluated in clinical trials for the treatment of acute kidney injury (9 registered) and liver injury (4 registered) (https://clinicaltrials.gov). Here, we show for the first time an attenuation of both liver and kidney damage in the context of HS. Nowadays, the driving mechanisms by which MSC moderate organ injury are still in debate. This could be either a direct effect of MSC, through antioxidant or trophic factors [31, 32]. Or it could be an indirect, immunomodulatory effect of MSC, through a downregulation of inflammation notably by reducing systemic cytokines [27, 33].

Systemic IL-6, IL-1α, and IL-10 cytokines were early upregulated in SIRS/CARS pathology [34, 35]. IL-6 is usually correlated with mortality and can be a used as biomarker to identify patients at risk of developing MODS after trauma [36]. The IL-1α precursor is constitutively present in epithelial layers (such as gastrointestinal tract, lung, liver, kidney, or endothelial cells) and is also produced by macrophages. When ischemia induces cell necrosis, a fully active IL-1α is released and functions as an “alarmin” which mediates the early phase of sterile inflammation. In contrast, IL-1β precursor is produced by hematopoietic cells in response TLR activation. Then, IL-1β precursor is activated mainly by caspase-1 cleavage which requires inflammasome activation (notably NLRP3) [37]. Both IL-1α and IL-1β exacerbate HS injury. Interestingly, IL-1α plays a critical role in cell death-induced inflammation and blocking its pathway could be an attractive strategy. For example, it was demonstrated that mice deficient for IL-R (IL-1α receptor) had significantly less hepatocyte damage in response to a toxic insult [38]. We observed that HS induced an upregulation of these pro and anti-inflammatory cytokines. Similar results were recently reported in mice, showing an increase in circulating IL-6 and IL-10 cytokine levels 90 min and 24 h after HS [39]. These results are consistent with the clinical literature, including a study on a cohort of 89 adult trauma patients that revealed a leukocytosis, an elevated serum pro- and anti-inflammatory cytokines, and evidence of innate cell activation, within minutes of trauma [26]. MSCp infusion decreased IL-1α, IL-6, and IL-10 plasma levels.

During sterile inflammatory response, granulocytes serve as first responders followed by monocytes. It was already described that circulating neutrophil number increased sharply at 3 h after injury and then decreased at 6 and 12 h, suggesting end organ sequestration. Moreover, 12 h after injury, the decrease in circulating neutrophils were significantly greater in MOF than in non-MOF patients [40]. Bone marrow monocytes that enter the bloodstream are thought to have an inflammatory phenotype and become the main source of inflammatory macrophages in tissues after trauma [36, 41]. Furthermore, a link between their expression of membrane markers and their secretion has been described. For example, CD80/86 signal via NF-κB pathway could induce the secretion of numerous cytokines, most notably IL-6 [42] and CD80/86 blockade could improve survival of septic mice and attenuate pro-inflammatory cytokine production [43]. Tissue damages are frequently observed if over-inflammation persists and is not adequately controlled. Monocytes/macrophages play an essential role in the complex and highly coordinated resolution phase. An increase in CD80 on monocytes and both CD80 and CD86 costimulatory receptors on granulocytes was observed in our study. After MSCp infusion, innate cells showed a lower expression of CD80 and CD86 compared to HS alone, suggesting a possible correlation with the decrease in pro-inflammatory cytokines, as suggested by Nolan et al., in 2008 [43]. As described in the introduction, HS can induce both an intense SIRS counterbalancing by CARS very early post-injury. PD-1 and PD-L1 molecules constitute a system of negative regulators involved in controlling T-cell responses. In sepsis, PD-L1 expression on monocytes is an important inducer of sepsis-induced immune alterations. In a polymicrobial sepsis model, PD-1 knockout in mice increases survival rate [8]. Moreover, correlations between increased monocyte PD-L1 expression and decreased expression of HLA-DR or in-vitro TNF-α release (both typical markers of monocyte alterations) has been described [44]. The regulation of the PD-1/PD-L1 axis is a very promising pathway in trauma [45] as it is in sepsis. The present study showed an increase PDL-1 on monocytes/granulocytes upon HS as reported in human [46] and MSCp reduced their expression. Our results are in agreement with the results obtained by Cohen et al. in a mouse model of HS. To characterize the impact of hemorrhage on myeloid cells, they observed an increased number of highly expressing PD-L1 myeloid cells in response to HS. They also observed a simultaneous induction of a hyperinflammatory state. They finally demonstrated that mice transplantation of neutrophil progenitors following HS decreases the proportion of host neutrophils with a suppressive phenotype and also attenuates their “priming” [39]. Therefore, MSCp therapy decreased both activation and inhibition markers on monocytes and granulocytes and the plasmatic expression of pro and anti-inflammatory cytokines. Altogether, our results suggest a return to homeostasis induced by MSCp in a dysregulated immune context.

In this report, IL-1β priming of MSC is necessary to obtain a therapeutic effect. IL-1β pro-inflammatory priming enhances MSC’s therapeutic activity through pro and anti-inflammatory mediators. A study of Amann et al. had already shown that the stimulation of MSC with IL-1β or sera from polytrauma patients tended toward an increase in pro-inflammatory, pro-angiogenic factors (IL-6, VEGFA), and chemokines (CXCL1, CCL2) in their supernatant. Their data also revealed that priming MSC with IL-1β may improve the therapeutic effect of MSC by induction of cell adhesion molecules and anti-inflammatory and anti-fibrotic molecules (ICAM1, MMP1, MMP10, IL1RN, TNFAIP6, VEGFA) [47]. In our study, IL-1β priming induced an upregulation of chemokine CCL2 or cytokine IL-6. CCL2 is one of the key chemokines that regulate migration and infiltration of monocytes/macrophages [48]. A recent study also demonstrated that MSC derived CCL2 is required for the interaction between MSC and macrophages. Indeed, MSC isolated from CCL2 deficient mice were unable to repolarize macrophages to the same extent as wild type-MSC. Therefore, CCL2 appears to have a potent effect on the ability to reduce the inflammatory response [49]. Moreover, it has also been demonstrated that IL-6 is a key cytokine for the immunoregulatory effects of MSC, through macrophages M2 polarization [50]. Similarly, we showed an increase of the anti-inflammatory lipid PGE2 in MSCp supernatant after priming. Németh et al. reported that MSC infusion can significantly improve animal survival following sepsis via monocytes/macrophages reprogramming by PGE2 secretion [51]. As our in vivo data suggested a role of monocytes in MSC-mediated immunomodulation, we performed a functional in vitro assay using monocytic line cells (THP-1). THP-1 stimulated by LPS tend toward a pro-inflammatory profile. We have shown that the coculture of LPS-treated THP-1 with naive MSC, but above all, with MSC primed by IL-1β, could inhibit the secretions of TNF-α and IL-6 produced by the THP-1 and promote the secretion of anti-inflammatory IL-1RA, showing that MSCp are able to modulate the response of activated monocytes. Collectively, these results demonstrate that IL-1β priming can enhance the therapeutic activity of MSC through both anti- and pro-inflammatory mediators. Therefore, therapeutic MSC seem to have a role in the orchestration of the inflammatory response by allowing the activation of immune cells in the inflammatory environment during the first phases [52, 53]. Then, a switch to an inhibitory role would be set up to avoid prolonged tissue damage. The mechanisms involved could be an attraction of immune cells via chemokines and a communication through bioactives molecules like IL-6 and PGE2 toward homeostasis state. The mechanism of the efficacy of MSC therapy remains a challenging question and requires further investigation.

## Conclusion

This work demonstrates that the early administration of rat IL-1β-primed bone marrow MSC could moderate HS-induced AKI and liver injury in a context of dysregulated systemic inflammation. These works further indicate that IL-1β priming is required to achieve a therapeutic efficiency of MSC. Our hypothesis is that early administration of MSCp could to modulate organ injury through attenuation of a complex immune dysfunction. Further studies are necessary to evaluate this therapeutic product in a context of traumatic hemorrhagic shock closer to the pathophysiological conditions encountered in the clinic.

## Supplementary Information

**Additional file 1: Fig S1.** Lung dysfunction.

## Data Availability

All data generated or analyzed during this study are included in this published article. These data are available from Juliette.peltzer@inserm.fr.

## Abbreviations

AKI: Acute kidney injury
ALT: Alanine aminotransferase
AST: Aspartate aminotransferase
BE: Base excess
bFGF: Basic fibroblast growth factor
BL: Baseline
BUN: Blood urea nitrogen
CARS: Compensatory anti-inflammatory response syndrome
CCL2: Chemokine ligand 2
CD: Cluster of differentiation
CXCL1: Chemokine (C-X-C motif) ligand 1
DAB labeling: 3,3′-Diaminobenzidine
DAMPs: Danger-associated molecular patterns
FCS: Fetal calf serum
FiO_2_: Fraction of inspired oxygen
GGT: Gamma-glutamyl transferase
HLA-DR: Human leukocyte antigen-DR isotype
HES: Hematoxylin-eosin-safran
hIL-1RA: Human interleukin-1 receptor antagonist
hIL-6: Human interleukin-6
hTNF-α: Human tumor necrosis factor alpha
HS: Hemorrhagic shock
Ht: Hematocrit
I/R: Ischemia/reperfusion
ICAM: Intercellular adhesion molecule
IL-1β: Interleukin-1 β
IL-10: Interleukin-10
IL-6: Interleukin-6
IL-1RA: Interleukin-1 receptor antagonist
IQR: Interquartile range
KIM-1: Kidney injury molecule-1
LPS: Lipopolysaccharide
MAP: Mean arterial pressure
MEMα: Minimum essential medium alpha
MHC II: Major histocompatibility complex II
MMP1: Matrix metalloproteinase-1
MMP10: Matrix metalloproteinase-10
MODS: Multiple organ dysfunction syndrome
MSC: Mesenchymal stromal cells
MSCn: Naive MSC
MSCp: Primed MSC
NF-κB: Nuclear factor kappa-light-chain-enhancer of activated B cells
PaCO_2_: Partial pressure of carbon dioxide
PaO_2_: Partial pressure of oxygen
PAS: Periodic acid-Schiff
PBS: Phosphate-buffered saline
PD-1: Program death-1
PD-L1: Program death-ligand 1
PGE2: Prostaglandine E2
SIRS: Systemic inflammatory response syndrome
tPO_2_: Tissue oxygen pressure
TNF-α: Tumor necrosis factor alpha
TNFAIP6: TNF alpha-induced protein 6
VEGFA: Vascular endothelial growth factor A
