# Supplementary material for: IL-1β primed mesenchymal stromal cells moderate hemorrhagic shock-induced organ injuries
Source: Stem Cell Res Ther. 2021 Aug 5;12:438. doi: 10.1186/s13287-021-02505-4 (PMC8340459; doi:10.1186/s13287-021-02505-4)
Supplement: Supplementary file 1 — Additional file 1: Fig S1. Lung dysfunction. [file 13287_2021_2505_MOESM1_ESM.docx]

**
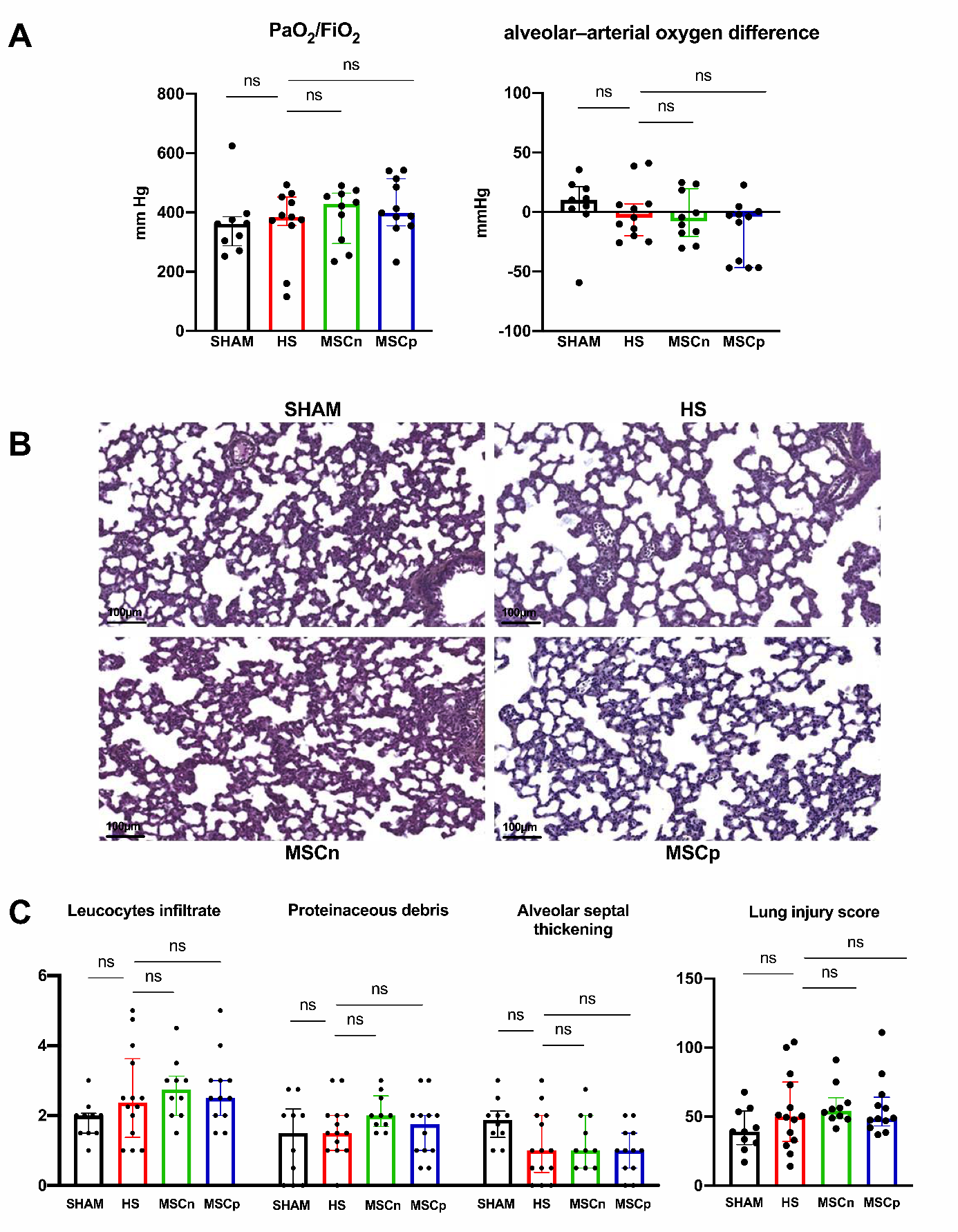
**

**Fig S1. Lung dysfunction**

**(A)** Ratio PaO_2_ / FiO_2_ and alveolar-arterial oxygen difference (n= 9, 11, 10 and 11 in Sham, HS, MSCn and MSCp groups respectively). **(B)** The lung was collected and stained by HES. Representative pictures were showed for every experimental group. **(C)** Lung injury scores (n= 10, 14, 10 and 12 in Sham, HS, MSCn and MSCp groups respectively). Data are expressed as median and interquartile range in the four experimental groups. Mann-Whitney statistical analysis was used to test the effect of our hemorrhaging model versus Sham. Dunn's test (with adjustment for multiplicity) was used to compare the effects of the treatments (MSCn and MSCp) to the HS group. HS: hemorrhagic shock, MSCn or MSCp: naive or primed mesenchymal stromal cells, PaO_2_: partial pressure of oxygen, FiO_2_: inspired oxygen faction, HES: Hematoxylin-Eosin-Safran.
